# Immunogenicity in Rabbits of Virus-Like Particles from a Contemporary Rabbit Haemorrhagic Disease Virus Type 2 (GI.2/RHDV2/b) Isolated in The Netherlands

**DOI:** 10.3390/v11060553

**Published:** 2019-06-14

**Authors:** Qiuhong Miao, Ruibing Qi, Luut Veldkamp, Jooske Ijzer, Marja L. Kik, Jie Zhu, Aoxing Tang, Dandan Dong, Yonghong Shi, Monique M. van Oers, Guangqing Liu, Gorben P. Pijlman

**Affiliations:** 1 Innovation Team of Small Animal Infectious Disease, Shanghai Veterinary Research Institute, Chinese Academy of Agricultural Science, Shanghai 200241, China; qiuhong.miao@wur.nl (Q.M.); gqyzqrb@gmail.com (R.Q.); zj121@shvri.ac.cn (J.Z.); tax1366@163.com (A.T.); dddong2015@163.com (D.D.); shiyonghong@shvri.ac.cn (Y.S.); 2 Laboratory of Virology, Wageningen University & Research, 6708 PB Wageningen, The Netherlands; luut.veldkamp@wur.nl (L.V.); monique.vanoers@wur.nl (M.M.v.O.); 3 Dutch Wildlife Health Centre (DWHC), 3584 CL Utrecht, The Netherlands; J.Ijzer@uu.nl (J.I.); m.kik@uu.nl (M.L.K.)

**Keywords:** rabbit haemorrhagic disease virus 2 (GI.2/RHDV2/b), Netherlands, VP60, baculovirus expression, virus-like particles, insect cells, immunogenicity

## Abstract

Rabbit haemorrhagic disease virus (RHDV) type 2 (GI.2/RHDV2/b) is an emerging pathogen in wild rabbits and in domestic rabbits vaccinated against RHDV (GI.1). Here we report the genome sequence of a contemporary RHDV2 isolate from the Netherlands and investigate the immunogenicity of virus-like particles (VLPs) produced in insect cells. RHDV2 RNA was isolated from the liver of a naturally infected wild rabbit and the complete viral genome sequence was assembled from sequenced RT-PCR products. Phylogenetic analysis based on the VP60 capsid gene demonstrated that the RHDV2 NL2016 isolate clustered with other contemporary RHDV2 strains. The VP60 gene was cloned in a baculovirus expression vector to produce VLPs in Sf9 insect cells. Density-gradient purified RHDV2 VLPs were visualized by transmission electron microscopy as spherical particles of around 30 nm in diameter with a morphology resembling authentic RHDV. Immunization of rabbits with RHDV2 VLPs resulted in high production of serum antibodies against VP60, and the production of cytokines (IFN-γ and IL-4) was significantly elevated in the immunized rabbits compared to the control group. The results demonstrate that the recombinant RHDV2 VLPs are highly immunogenic and may find applications in serological detection assays and might be further developed as a vaccine candidate to protect domestic rabbits against RHDV2 infection.

## 1. Introduction

Rabbit haemorrhagic disease virus (RHDV, family Caliciviridae, genus *Lagovirus*) was first reported in 1984 in China as the causal agent of Rabbit Haemorrhagic Disease (RHD), an acute and highly lethal disease in wild and domestic rabbits (*Oryctolagus cuniculus*). Two years after the initial outbreak, RHDV reached the European continent. RHDV is highly contagious and can have a mortality rate of around 80–100% in adult rabbits [1]. RHD is considered the most economically important disease in rabbits around the world [2]. Several inactivated virus and live myxoma vector vaccines are commercially available that confer protection against circulating RHDV strains [1]. Even after large scale vaccination numerous cases of RHD remained to be reported, e.g., in 2010 in north-western France [3]. This disease affected not only wild populations, but also RHDV-vaccinated rabbits, suggesting the emergence of a novel RHDV serotype. Indeed, a newly identified lagovirus named RHDV type 2 (RHDV2) was identified on an industrial farm in the province of Udine in north-eastern Italy [4]. Later, a similar virus isolated in Spain was named RHDVb [5]. In 2017, a new RHDV nomenclature was proposed and RHDV2 and RHDVb were designated GI.2 [6]. RHDV2 (RHDVb/GI.2) has been reported in France [3], Italy [7], the Iberian Peninsula [8,9], Sardinia [10], the United Kingdom [11], Madeira [12], the Canary Islands, the Azores [13], Australia [14], Scandinavia [15], Africa [16] and America [17]. In Sweden, Australia and on the Iberian Peninsula, all recent cases of RHD in wild and domestic rabbits were caused by RHDV2, which suggests that RHDV2 replaces RHDV as the main cause of RHD [8,18,19,20]. These outbreaks clearly indicated that RHDV2 is antigenically different from RHDV and has the capacity to kill rabbits vaccinated against RHDV [20,21].

In the period December 2015 to August 2016, the Dutch Wildlife Health Centre (DWHC) in the Netherlands examined numerous dead rabbits that were found to be RHDV2-positive. In January 2017, the first case of RHDV2 in the Netherlands was confirmed as the cause of death in a wild hare. The dead hare presented with severe acute inflammation in the liver and spleen, and RHDV2 infection was confirmed by PCR analysis.

At present, the emergence of RHDV2 raises concerns regarding the impact of the disease among domestic and wild rabbit populations. RHDV2 is replacing existing strains of RHDV and is expected to cause severe problems to the rabbit industry. Thus, there is a clear need for a safe and effective RHDV2 vaccine. The traditional approach is an inactivated vaccine, typically produced from the liver collected from artificially infected rabbits. Because inactivated RHDV2 vaccines may not always confer full protection against RHD, alternative vaccines including those based on a myxoma virus vector are in development. Another promising approach to develop safe and effective vaccines is the application of virus-like particles (VLPs). VLPs have been shown to induce a potent B-cell mediated immune response, and can induce highly effective CD4+ T cell proliferative responses and a cytotoxic T lymphocyte (CTL) response [22,23,24,25,26]. VLP-based vaccines have been recognized as safe and effective and are commercially available to protect humans against Hepatitis B virus and human papillomavirus [27,28] and swine against porcine circovirus [29]. Numerous prototype VLP vaccines against novel emerging diseases, such as chikungunya and Zika viruses have been generated and are in different stages of (pre-)clinical trials [30,31]. RHDV VLPs can be generated by overexpression and self-assembly of VP60, the major viral structural protein, using different expression systems including bacteria, yeast and insect cells [32,33,34,35,36,37,38]. The baculovirus expression vector system (BEVS) is a versatile expression platform ideally suited for the large-scale production of complex (glyco)proteins and VLPs in insect cells [39,40]. Commercial VLP-based vaccines for human (Cervarix, GlaxoSmithKline) and veterinary (Porcilis PCV, MSD Animal Health) applications utilize BEVS technology.

In the present study, we identified the complete genome sequence of a contemporary RHDV2 strain from the Netherlands (NL2016) and expressed the VP60 capsid protein of RHDV2-NL2016 to generate VLPs. Subsequently, we have evaluated the immunogenicity of the RHDV2 VLPs in rabbits.

## 2. Materials and Methods

### 2.1. Ethics Statement

All animal experiments involved in these studies were conducted following the guide for care and use of laboratory animals of the Ministry of Science and Technology of the People’s Republic of China. All experiments were performed in a secondary biosecurity laboratory. All animal procedures were approved by the Institutional Animal Care and Use committee of the Shanghai Veterinary Research Institute, Chinese Academy of Agricultural Sciences (permit number: 20180511). Every effort was made to minimize suffering.

### 2.2. RHDV2 Virus Sequence Analysis and Phylogenetic Analysis

RHDV2 was discovered by the Dutch Wildlife Health Centre in the Netherlands. The liver of the infected rabbit (*O. cuniculus*, no. 3151221028) was ground with liquid nitrogen in a mortar and total RNA was isolated with Trizol (Invitrogen, Carlsbad, CA, USA). Primers (Table 1) were designed based on conserved regions of RHDV2 sequences available in GenBank (Table 2).

Reverse transcription of total RNA with M-MLV reverse transcriptase and random primers was used to generate cDNA. The cDNA was used as template for PCR amplifications using primers (Table 1) to obtain a collection of DNA fragments spanning the entire RHDV2 genome. For these PCR amplifications the proof-reading Phusion polymerase (Thermo Fisher, Waltham, MA, USA) was used. Each PCR product was cloned into pJET1.2 (Thermo Fisher, USA) and 3 clones per fragment were sent for Sanger sequencing (Macrogen, Amsterdam, Netherlands). The sequences were assembled into a consensus complete genome sequence named *Lagovirus europaeus*/GI.2/*O cun*/NL/2016/3151221028, or in short RHDV2-NL2016 (Genbank accession no. MN061492). The phylogenetic tree was constructed by Mega5.1 using a selection of VP60 sequences (Table 2).

### 2.3. RHDV2 VP60 Cloning and Expression Vector Construction

Primers with attB1/2 recombination sites and *Nco*I and *Nsi*I unique restriction sites were designed to subclone the RHDV2 VP60 gene in the Gateway donor vector pDONR207 (Invitrogen, USA). Next, the VP60 gene was transferred to the Gateway destination vector pDEST10 (Invitrogen, USA), which drives expression of N-terminal his-tagged transgenes from the strong baculovirus polyhedrin promoter. The resulting pDEST10-VP60 plasmid was sequenced and used for the construction of a recombinant baculovirus.

### 2.4. Cells and Recombinant Baculovirus Expressing RHDV2 VLPs

*Spodoptera frugiperda* Sf9 insect cells were maintained in Sf-900ⅡSFM medium (Gibco, MA, USA) at 28 °C. The plasmid pDEST10-VP60 was transformed into DH10Bac *Escherichia coli* competent cells, to generate recombinant bacmid DNA (Bac-to-bac expression system, Invitrogen, USA). The recombinant bacmid DNA was used to transfect Sf9 insect cells to generate the corresponding recombinant baculovirus, Bac-RHDV2-VP60, using Cellfectin II transfection reagent (Invitrogen, USA). Viral titres were determined by an end-point dilution assay (EPDA) using Sf9 cells. Subsequent infections were performed by adding virus to cells at a multiplicity of infection (MOI) of 10 TCID_50_ units per cell. Cells were incubated for 2 h and then the medium was changed by fresh medium. Plaque purification was performed to isolate a pure viral stock. Briefly, the Sf9 cell monolayers were incubated with 10-fold serial dilutions of baculovirus Bac-RHDV2-VP60. After infection for 2 h, the medium containing virus was replaced by fresh medium, and the cells were overlaid with 1% low melting-point agarose (Sangon, ShangHai, China) in an Sf-900™ Medium (1.3×) medium (Gibco, USA) and incubated at 28 °C. After 7 days, several plaques were randomly picked from serial dilutions. Later the recombinant baculoviruses from individual plaques were amplified, titrated and then used to inoculate Sf9 cells.

### 2.5. Immunofluorescence Assay and Western Blot Analysis

Sf9 cells were infected with Bac-RHDV2-VP60 at a multiplicity of infection (MOI) of 10 followed by incubation at 28 °C for three days. Indirect immunofluorescence assay (IFA) was used to check the expression of VP60 in Sf9 cells. Sf9 cells were fixed with 4% paraformaldehyde, then an anti-His mouse monoclonal antibody was used for the first antibody and a FITC-conjugated rabbit-anti-mouse antibody was used for detection. To check the expression by western blot analysis, supernatants and cell lysates were collected. Cells were lysed in RIPA buffer (Beyotime, Shanghai, China) and anti His-tag antibodies were used for VP60 detection. Furthermore, Sf9 cells were infected with Bac-RHDV2-VP60 at an MOI of 10 to test the VP60 expression level at different timepoints post infection. Culture fluids and cells were collected from six-well plates, in volumes of 2 mL, and lysed by sonication with a 2 mm Ultrasonic Amplitude Transformer giving 5 s pulses at 300W with intervals of 9 s for a total of 10 min. Next, the lysates were clarified by centrifugation at 8000 rpm for 15 min to remove the cell debris. Then, the lysates were subjected to ultracentrifugation at 40,000 rpm for 3 h to pellet the VLPs. Finally, the pellets were resuspended in 80 μL PBS and analysed by SDS-PAGE and western blot analysis.

### 2.6. Transmission Electron Microscopy

To test the ability of RHDV2 VP60 to self-assemble into VLPs, the supernatants of lysed Bac-RHDV2-VP60 infected Sf9 cells were clarified and VLPs were pelleted as above and then subjected to caesium chloride density gradient centrifugation. The VP60 band was isolated with a needle, diluted in PBS and centrifuged again to remove the CsCl. Finally, the pellet was resuspended in PBS. The caesium chloride purified material was checked by TEM via negative staining with 0.5% aqueous uranyl acetate. The grids were observed with a transmission electron microscope (H-7500, Hitachi, Japan) operating at 80 kV.

### 2.7. Preparation of RHDV2 VLPs and Control Antigen

To prepare RHDV2 VLPs for immunization trials, Sf9 cells were infected at an MOI of 1 for 72 h with plaque-purified Bac-RHDV2-VP60. Cells were lysed ultrasonically in the growth medium, and then clarified by centrifugation at 8000 rpm for 15 min. The supernatants were collected and the total protein concentration was measured by a BCA assay and then used for immunization experiments. To formulate the experimental vaccines, MONTANIDE ISA 201VG adjuvant (SEPPIC, Shanghai, China) was mixed at a ratio of 1:1 with VLPs by Emulsifying Equipment (FLUKO, Shanghai, China) for injection. Sf9 cell lysates from uninfected cells were subjected to the same treatments to generate the negative control antigen.

### 2.8. Vaccination of Rabbits with RHDV2 VLPs and ELISA Assays

Fifteen 8-week-old New Zealand White Rabbits which lacked anti-RHDV antibodies were purchased from the Laboratory Animal Centre of the Shanghai Veterinary Research Institute and raised in pathogen-free, isolated cages. All animals used in the immunization trials were approved in compliance with the guidelines of the Animal Research Ethics Board of the Shanghai Veterinary Research Institute, Chinese Academy of Agricultural Sciences (CAAS). Rabbits were immunized with 2 mg VLPs, 5 mg VLPs or with control antigen via subcutaneous injection. Serum samples were collected from each immunized rabbit at 0, 7, 14 and 21 days after immunization. RHDV2-specific antibodies in immunized rabbits were detected by indirect ELISA assays as described [33]. Briefly, 96-well microplates were coated overnight at 4 °C, with GST-tagged RHDV2 VP60 protein produced in *E. coli* using a PGEX-4T-1 expression vector, followed by blocking the plates with 5% skimmed milk diluted in PBS-Tween (PBST) at 37 °C for 1 h. After washing with PBST, the plates were incubated for 1 h with 100 μL of serum samples (1:500 diluted in PBS), and then washed with PBST. Next, 100 μL of HRP-conjugated goat anti-rabbit IgG (Jackson ImmunoResearch, West Grove, PA, USA) was added at a dilution of 1:10,000 and again incubated for 1 h. The plates were washed three times and then 100 μL TMB substrate (Beyotime, China) was added and the plate was incubated in the dark for 15 min. Finally, 50 μL 2 M sulfuric acid was added to stop the colour development, after which the absorbance was measured at 450 nm by using a microplate reader (Biotek, Winooski, VT, USA). All samples obtained were measured in quadruplicate.

### 2.9. Cytokine Assays

The level of cytokines at weeks 0, 1, 2 and 3 post-immunization was examined to investigate the efficiency of the cellular immune response induced by VLPs. Therefore, the serum collected above was used to determine the levels of the Th1-Type I related cytokine IFN-γ, while IL-4 levels were measured to check for Th2-type 2 cytokines. Commercial Elisa Kits (R&D Minneapolis, MN, USA) were used for these assays.

### 2.10. Statistical Analysis

All statistical analyses were performed using GraphPad Prism Version 5. The data including antibody response and cytokine production were compared by one-way ANOVA among different groups. A *p* < 0.05 was considered significantly different.

## 3. Results

### 3.1. Pathology of RHDV2 Infection in a European Rabbit

A specimen of the European rabbit (*O. cuniculus*) was found dead on 22 December 2015 in a garden in Nederweert, in the province of Limburg in the Netherlands. Post-mortem examination showed it was a young adult male, in good condition (based on muscle development and degree of fat storage). The trachea mucosa was hyperaemic, and the anterior lung lobes were dark red and consolidated. The liver was light brown and soft. The gastrointestinal tract was filled with feed remains, and a few coccidia and cestode and nematode parasites were observed (incidental findings). Main histopathologic findings were diffuse swollen and hyper-eosinophilic hepatocytes, diffuse hyperaemia and necrosis of lymphoid cells in the spleen, and pulmonary hyperaemia with moderate to severe intra-alveolar oedema and focal haemorrhage. The observed per acute hepatic necrosis, splenic necrosis and acute pneumonia are fatal lesions consistent with RHDV2 infection, which was confirmed by PCR analysis.

### 3.2. Complete Genome Sequence and Phylogeny of RHDV2 Isolate NL2016

The liver of the dead rabbit was collected and used for isolation of total RNA. As the 5′UTR of RHDV2 is conserved among different strains, primers for RT-PCR were designed based on the conserved regions of RHDV2, while for the 3′UTR we designed a primer ending with oligo-dT (Table 1). RT-PCR was performed, and the amplified PCR products were cloned into pJET1.2 and then processed for Sanger sequencing. The sequences were assembled into a consensus complete RHDV2 genome (named NL2016 isolate) with a length of 7473 nucleotides (nt) (Genbank accession no. MN061492).

**Figure 1:**
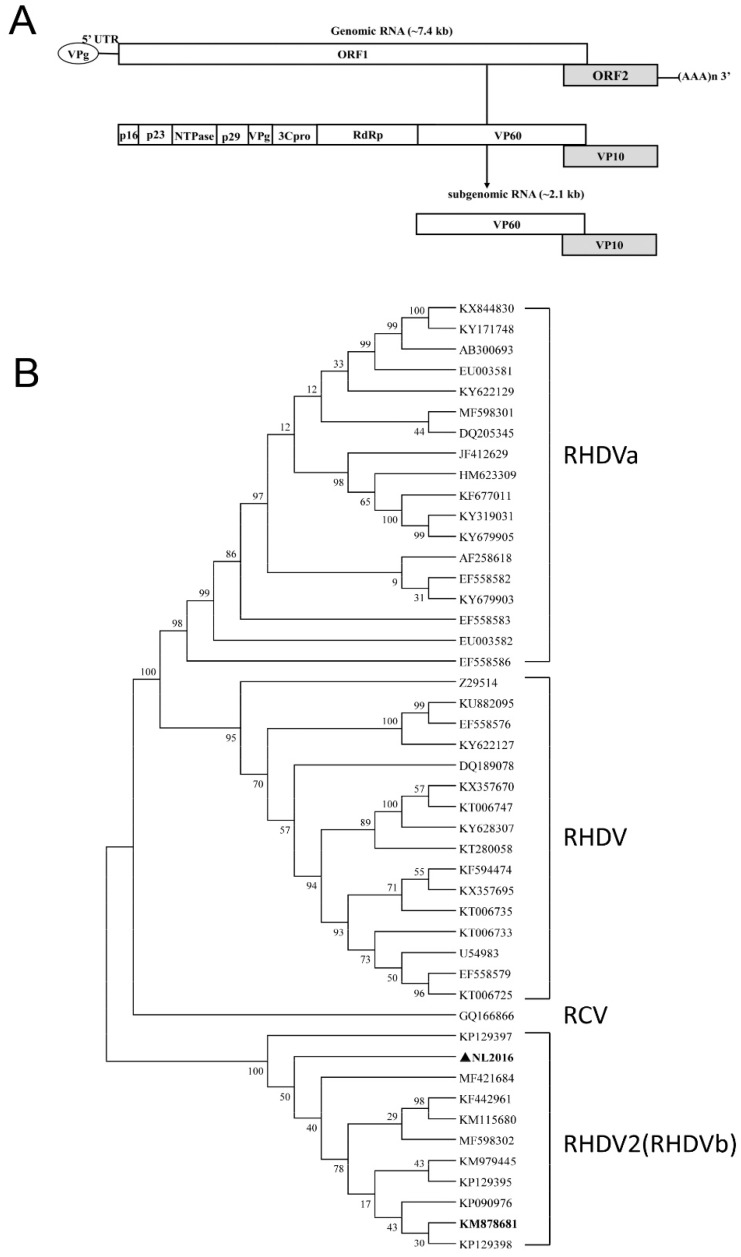
Genome organization of strain RHDV2-NL2016 and phylogenetic analysis. (**A**) Genome organization of RHDV2. The RHDV2 genome is a 3′polyadenylated single-stranded positive-sense RNA of ~7.4 kb encoding 2 open reading frames (ORFs). A subgenomic mRNA of ~2.1 kb is formed during viral RNA replication. A viral genome-linked protein (VPg) is covalently attached to the 5′ untranslated region (5′UTR). ORF1 encodes a polyprotein that is proteolytically cleaved to form the non-structural proteins p16, p23, NTPase, p29, VPg, the viral protease 3Cpro, the RNA-dependent RNA polymerase (RdRp) and the major capsid protein VP60. ORF2 encodes the minor structural protein VP10. (**B**) Phylogenetic neighbour-joining tree constructed based on the VP60 gene of RHDV2-NL2016 and multiple reference sequences (Table 2).

The RHDV2-NL2016 genome has two opening reading frames (ORFs) (Figure 1A), of which the first ORF is processed in a number of non-structural proteins and the structural protein VP60. By selecting RHDV and RHDV2 GenBank sequences (Table 2) we constructed a phylogenetic tree based on the VP60 nucleotide sequences. The NL2016 isolate clustered with other RHDV2 (RHDVb) isolates including the type strain from Spain (RHDV2-N11, GenBank: KM878681.1). This indicates that NL2016 is a typical RHDV2 strain (Figure 1B).

### 3.3. Baculovirus Expression of RHDV2 VP60 in Sf9 Insect Cells

In order to produce RHDV2 VLPs in insect cells, the VP60 ORF was PCR amplified and cloned downstream of a His-tag into the expression plasmid pDEST10 under control of the very strong polyhedrin promoter. The pDEST10-VP60 plasmid was used to construct a recombinant baculovirus bacmid, which was then transfected into Sf9 insect cells to generate the recombinant baculovirus Bac-RHDV2-VP60.

**Figure 2:**
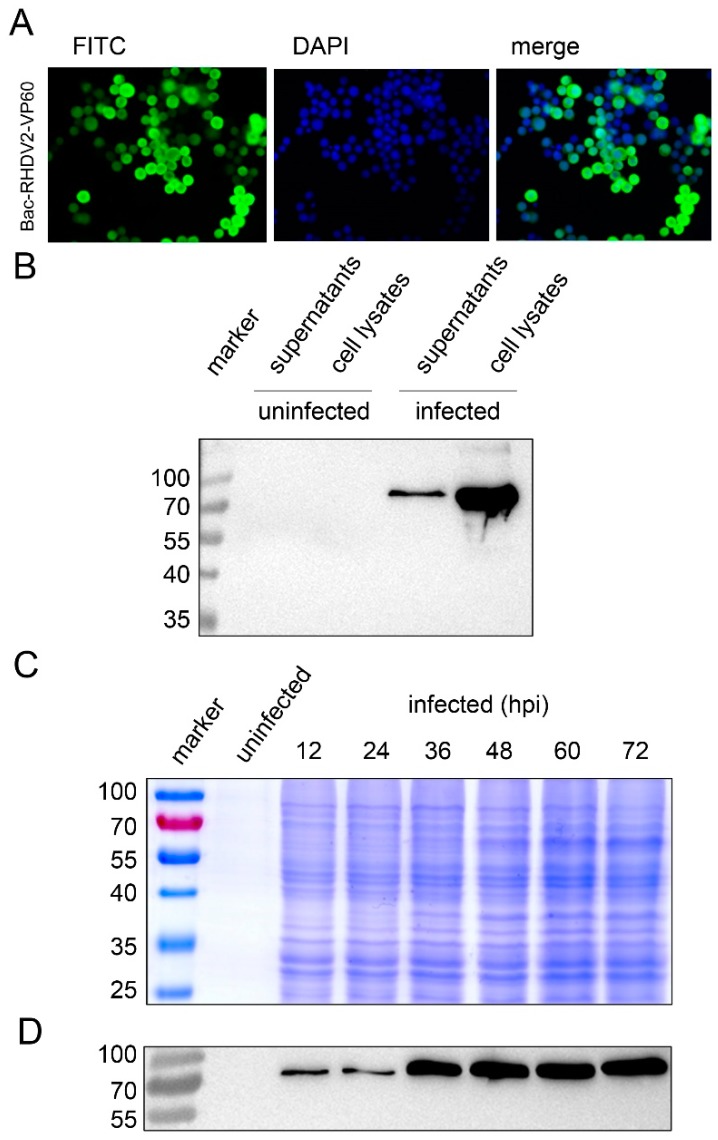
VP60 synthesis by infection of Sf9 cells with a recombinant baculovirus. (**A**) Indirect immunofluorescent assay to show the expression of His-VP60 in transfected Sf9 cells. Anti-His mAb was used as the primary antibody, and a FITC-conjugated rabbit-anti-mouse antibody was used for detection. DAPI was used to stain cell nuclei (200× magnification). (**B**) Western blot (WB) analysis of Bac-His-VP60 infected Sf9 cells. Cells were infected with the P5 baculovirus stock at a multiplicity of infection (MOI) of 10, collected at 3 days post infection (dpi) and checked by WB. Anti-His mAb was used as the primary antibody, and an HRP-conjugated rabbit-anti-mouse antibody was used for detection. (**C**) SDS-PAGE and (**D**) WB were used to check the expression level of His-VP60 in Sf9 cells at different times point infection. Anti-His mAb was used as the primary antibody, and an HRP-conjugated rabbit-anti-mouse antibody was used for detection.

An indirect immunofluorescence assay (IFA) using anti-his antibodies showed that VP60 accumulated to high levels upon transfection of bacmid DNA (Figure 2A). Supernatants and lysates of infected cells were separately collected after 3 days post infection (dpi) with plaque purified, passage 5 (P5) virus stocks. Next, VP60 expression was confirmed by western blot analysis using His-tag specific antibodies. The expected molecular mass of recombinant His-VP60 is approximately 70 kDa (Figure 2B). Both the supernatant and cell lysates were positive, with more protein present in the cell lysate. Next, Sf9 cells infected with Bac-RHDV2-VP60 at an MOI of 10 and both the culture fluid and cells were collected, lysed by sonication and clarified by low-speed centrifugation to remove cell debris. After ultracentrifugation, the pellet was resuspended in PBS and the expression level of RHDV2-VLPs was checked by SDS-PAGE (Figure 2C) and western blot (Figure 2D). RHDV2-VLPs could be detected on western blots as early as 12 hpi (Figure 2D), but the highest expression level was observed at 60 hpi (Figure 2C).

### 3.4. Characterization of RHDV2 VLPs by Electron Microscopy

**Figure 3:**
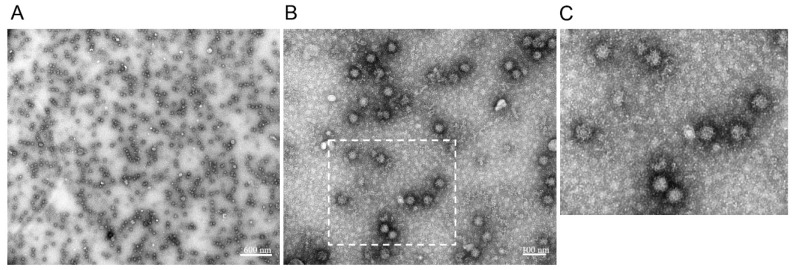
TEM pictures of rabbit haemorrhagic disease virus type 2 virus-like particles (RHDV2 VLPs) at different magnifications. Transmission electron microscopy of CsCl density gradient purified RHDV2 VLPs. Samples were negatively stained with 0.5% aqueous uranyl acetate. Bars represent 600 nm (**A**) and 100 nm ((**B**), inset enlarged in (**C**)).

To determine whether the overexpressed RHDV2 VP60 protein self-assembled into VLPs, CsCl density gradient purified material was visualized by electron microscopy analysis. TEM showed a high concentration of spherical particles (Figure 3A) with diameters ranging from 30–35 nm (Figure 3B,C). This demonstrates the successful assembly of RHDV2 VLPs in Sf9 insect cells.

### 3.5. Immunogenicity of RHDV2 VLPs in Rabbits

**Figure 4:**
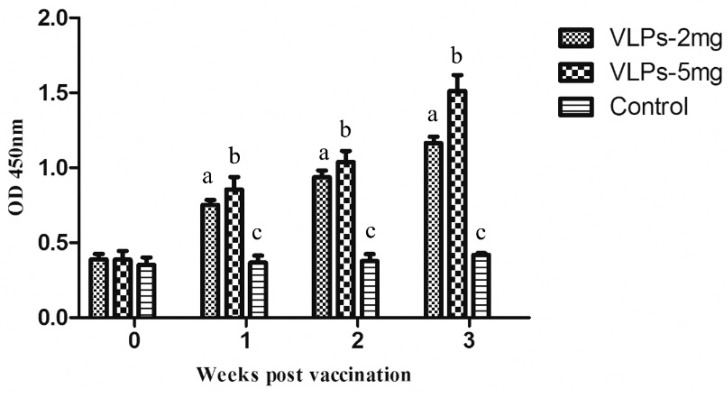
Serum antibody responses in rabbits after immunization with RHDV2 VLPs. Rabbit sera from different groups of immunized animals were collected at 0, 1, 2 and 3 weeks post immunization and then analysed by indirect ELISA. All rabbit sera were diluted to 1:500 before testing.

The RHDV2 VLPs were then used to immunize rabbits. Groups of five rabbits were immunized subcutaneously with different dosages of VLPs or with control antigen. Serum was collected every week and RHDV2-specific antibodies in immunized rabbits were detected by indirect ELISA assays. The antibody titers of rabbits immunized with VLPs were shown to increase with time and are statistically different from the control group. As expected, the antibody titers in rabbits immunized with 5 mg VLPs are a little higher than those immunized with 2 mg VLPs (Figure 4).

**Figure 5:**
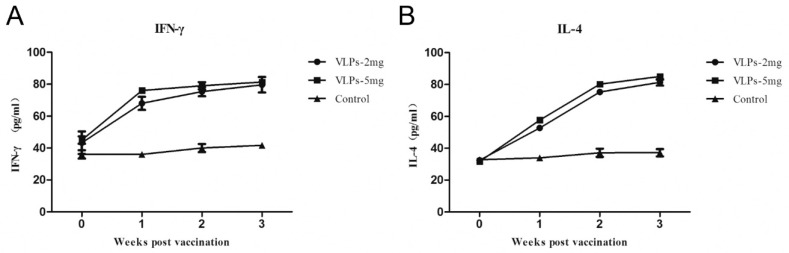
Production of cytokines IFN-γ (**A**) and IL-4 (**B**) in rabbits after immunization. The rabbit sera collected were used to analyse the serum levels of IFN-γ and IL-4 by ELISA. The IFN-γ and IL-4 level of rabbits immunized subcutaneously with 2 mg or 5 mg VLPs were both significantly higher as compared to the control group, but not different from each other.

To investigate the cellular immune response to RHDV2 VLP immunization, IFN-γ and IL-4 production levels were measured in sera from immunized rabbits. The levels of IFN-γ (Figure 5A) and IL-4 (Figure 5B) in both VLP groups were increasing over time and were significantly different from the control group, indicating that both Th1 and Th2 responses were induced by the VLPs.

## 4. Discussion

RHD is a highly contagious and lethal viral disease of both wild and domestic rabbits and associated with large economic losses. RHD may be caused by related but different lagoviruses. Since the first discovery of RHDV2 in 2010 in RHDV-vaccinated rabbits, RHDV2 has spread through wild rabbit populations and has replaced RHDV in several countries. RHDV2 is genetically and antigenically distinct from RHDV and thus can overcome immunity to classical RHDV. The case-fatality in mature rabbits infected with RHDV2 is lower compared to classic RHDV infection, however, young rabbits are more susceptible to RHDV2 [21]. Before GI.2 emerged, only *Lepus granatensis* [41] and *O. cuniculus* had been infected with RHDV. With the emerge of GI.2, more species have been observed to become infected, e.g., *Lepus corsicanus* [42], *Lepus europaeus* [43,44], *Lepus capensis mediterraneus* [10] and *Lepus timidus* [45]. Thus, it is urgent to control and prevent transmission of RHDV2.

Traditional RHD inactivated vaccines are effective, but production of the viral antigens can be cumbersome given the lack of cell lines supporting RHDV and RHDV2 infection. VLPs are a product of modern biotechnology and can be produced at large scale, at low cost, and generally at the lowest biosafety level. VLPs structurally resemble the authentic virus as they are the result of self-assembly of solely the viral structural protein(s). Due to the lack of viral genetic material inside the capsid, VLPs are non-infectious and have a similar safety profile as subunit vaccines. However, in comparison to subunits, VLPs display their antigenic epitopes in a structured, repetitive fashion that may serve as pathogen-associated molecular patterns (PAMPs). In this way, co-stimulatory signals are available for the activation of T-lymphocytes by antigen presenting cells. This is one of the reasons that VLPs in general tend to generate a more potent humoral and cellular immune response [46].

RHDV VLPs have been produced in multiple expression systems, including the BEVS. The advantage of baculovirus expression in insect cells is the ease of use and robustness of the system, leading to high expression of almost any pro- or eukaryotic protein. Another advantage of the BEVS is the efficiency of post-translation modifications, such as phosphorylation or glycosylation, which is important for the functionality of complex viral (glyco)proteins [39]. Indeed, it has been shown that RHDV VP60 capsid protein expression in insect cells leads to the formation of VLPs that can easily be purified for downstream applications [47]. VLPs from new variant RHDV2(GI.2) have been generated to demonstrate the different antigenic properties exhibited by RHDV and RHDV2 [48], to generate monoclonal antibodies against the RHDV2 capsid, to evaluate the sensitivity and specificity of an ELISA for the detection of RHDV2 in liver extracts [49] and to test their immunogenicity and protection potential against a lethal virus challenge [50].

In our study, we obtained the complete RHDV2 genome sequence from a diseased rabbit, which was found in the Netherlands in 2016. The contemporary RHDV2-NL2016 isolate appeared to be closely related to other European RHDV2 genotypes for which the VP60 sequence is available, suggesting that the virus quickly spread over the continent after its initial introduction. It is known that RHDV2 strains have evolved by recombination between the non-structural proteins and minor structural proteins, VP60 and VP10. Recombinants may, e.g., include the structural proteins of GI.2, with non-structural proteins derived from non-pathogenic lagoviruses (GI.3 and GI.4) or from pathogenic GI.1 strains [51,52]. However, according to the phylogenetic tree we generated based on the amino acid sequence of the NL2016 non-structural proteins, we could only see that NL2016 shares a close relationship with other RHDV2(RHDVb/GI.2) sequences (KM979445, KP129397) and not with GI.1 strains.

We cloned the RHDV2-NL2016 VP60 gene in a recombinant baculovirus expression vector using Gateway and Bac-to-bac technology and demonstrated the successful expression of RHDV2 VLPs in Sf9 insect cells (Figure 2 and Figure 3). The icosahedral VLPs had a regular, spherical appearance. The VLPs could easily be purified and were indistinguishable from infectious virus when visualized by electron microscopy. Immunization of rabbits with RHDV2 VLPs induced high levels of VP60-specific antibodies. Furthermore, the production of the Th1-Type I cytokine IFN-γ and the Th2-type 2 cytokine IL-4 in the immunized rabbits were significantly higher than in the negative control group.

Together, our results suggest that RHDV2 VLPs produced in insect cells with recombinant baculovirus technology are highly immunogenic in rabbits. The VLPs may be further developed as a promising, novel vaccine to protect rabbits against RHDV2. An attractive possibility would be to generate a bivalent RHD vaccine by combining the RHDV and RHDV2 VLPs in a single formulation. Finally, the VLPs may find application as a potent antigen in diagnostic serological assays to discriminate RHDV from RHDV2 infections.

## Figures and Tables

**Table 1:** Primers designed for amplification of the complete genome of a rabbit haemorrhagic disease virus type 2 strain from the Netherlands (RHDV2-NL2016).

Primer Name	Primer Sequence
Gate-VP60F(*NcoI*)	GGGGACAAGTTTGTACAAAAAAGCAGGCTTACCATGGACCATGGAGGGCAAAGCCCG
Gate-VP60R(*NsiI*)	GGGGACCACTTTGTACAAGAAAGCTGGGTAATGCATTCAGACATAAGAAAAGCCATTG
RHDV2(1-405)-F	GTGAAAGTTATGGCGGCTATG
RHDV2(1-405)-R	TCGGTAAGCACAGGGGATGAC
RHDV2(80-1355)-F	TCCTGGACCTCAGGGACAAGA
RHDV2(80-1355)-R	GCCATTTTCACAACTGTCAT
RHDV2(1332-3101)-F	GGTTATGACAGTTGTGAAAATGGC
RHDV2(1332-3101)-R	GTCATGTCATGTGCGTTGACA
RHDV2(3083-5319)-F	TCAACGCACATGACATGACTG
RHDV2(3083-5319)-R	GGCTTTGCCCTCCATAACATT
RHDV2(6964-7378)-F	CGCCCTGTGGGACCCAGA
RHDV2(6964-7378)-R	TCAAGCACTGGACTCGCCAGT
RHDV2(5295-7047)-F	TGTGAATGTTATGGAGGGCAAAGC
RHDV2- 3′dTNNN	GACTGACTGCCATGGCCGGCGCTAGCTTTTTTTTTTTTTTTTTTTTTTTTT

**Table 2:** Selected RHDV sequences to construct a phylogenetic tree.

Genbank Accession Number	Genotype	Strain Name	Year of Isolation
KX844830	RHDVa	SCH04	2016
KY171748	RHDVa	Sch07	2017
AB300693	RHDVa	Hokkaido/2002/JPN	2009
EU003581	RHDVa	NY-01	2007
KY622129	RHDVa	P175	2017
MF598301	RHDVa	K5_08Q712_BatchRelease1/2008	2017
DQ205345	RHDVa	JX/CHA/97	1997
JF412629	RHDVa	HYD	2011
HM623309	RHDVa	NJ-2009	2009
KF677011	RHDVa	STR2012	2012
KY679905	RHDVa	STR2014	2014
AF258618	RHDVa	Iowa2000	2000
EF558583	RHDVa	Triptis	2008
EU003582	RHDVa	UT-01	2001
EF558586	RHDVa	Hartmannsdorf	2007
Z29514	RHDV	SD	2005
KU882095	RHDV	ZD0	2000
EF558576	RHDV	Jena	2007
KY622127	RHDV	P158	1998
DQ189078	RHDV	Saudi Arabia	2005
KX357670	RHDV	AUS/ACT/MtPt-2/2010	2010
KT006735	RHDV	AUS/WA/Bunbury/2000	2000
KT006733	RHDV	AUS/WA/Gnowangerup/1999	1999
U54983	RHDV	Czech strain V351	1997
EF558579	RHDV	NZ54	2007
KT006725	RHDV	NZL/Otago/Queensberry/74/2013	2013
GQ166866	RCV	MRCV	2001
KP129397	RHDV2(RHDVb)	Tar06-12	2015
MF421684	RHDV2(RHDVb)	AUS/VIC/AC-1/2016	2016
KF442961	RHDV2(RHDVb)	Algarve1	2013
KM115680	RHDV2(RHDVb)	CBEstoi13-7	2013
MF598302	RHDV2(RHDVb)	AUS/NSW/CAR-3/2016	2016
KM979445	RHDV2(RHDVb)	CBVal16	2012
KP129395	RHDV2(RHDVb)	Rij06-12	2014
KP090976	RHDV2(RHDVb)	CBAnd1	2012
KM878681	RHDV2(RHDVb)	N11	2011
KP129398	RHDV2(RHDVb)	Zar11-11	2010
